# The best of both worlds: A combined approach for analyzing microalgal diversity via metabarcoding and morphology-based methods

**DOI:** 10.1371/journal.pone.0172808

**Published:** 2017-02-24

**Authors:** Sophie Groendahl, Maria Kahlert, Patrick Fink

**Affiliations:** 1 University of Cologne, Zoological Institute, Aquatic Chemical Ecology, Cologne, Germany; 2 Swedish University of Agricultural Sciences, Department of Aquatic Sciences and Assessment, Uppsala, Sweden; 3 University of Duesseldorf, Institute for Cell Biology and Zoology, Duesseldorf, Germany; University of Hyogo, JAPAN

## Abstract

An increasing number of studies use next generation sequencing (NGS) to analyze complex communities, but is the method sensitive enough when it comes to identification and quantification of species? We compared NGS with morphology-based identification methods in an analysis of microalgal (periphyton) communities. We conducted a mesocosm experiment in which we allowed two benthic grazer species to feed upon benthic biofilms, which resulted in altered periphyton communities. Morphology-based identification and 454 (Roche) pyrosequencing of the V4 region in the small ribosomal unit (18S) rDNA gene were used to investigate the community change caused by grazing. Both the NGS-based data and the morphology-based method detected a marked shift in the biofilm composition, though the two methods varied strongly in their abilities to detect and quantify specific taxa, and neither method was able to detect all species in the biofilms. For quantitative analysis, we therefore recommend using both metabarcoding and microscopic identification when assessing the community composition of eukaryotic microorganisms.

## Introduction

During the last century biodiversity has declined due to anthropogenic influences [1]. This results in reduced ecosystem stability, functioning and provision of ecosystem services [2–4]. While loss of biodiversity in larger organisms is well studied [1], we know far less about the effects of biodiversity loss for microorganisms such as algae. As microalgae are the base of aquatic food webs [5, 6], a reduction in algal diversity may have great repercussions for higher trophic levels. During the last century, most studies on the diversity, distribution, and abundance of algae were based on morphological characteristics e.g. [7–9]. However, this is a challenging and time-consuming method, since microalgal samples typically contain many cryptic, small, and rare species. Therefore considerable taxonomic expertise are required, which are unfortunately becoming increasingly rare [10]. The global number of algal species is estimated to be over a million species [11]. A conservative approach anticipated 72,500 algal species, of which only 60% have been described to date [11]. In order to quantify and to reduce the decline in biodiversity, we need more precise assessments of the current biodiversity. A newly developed method, called DNA metabarcoding, via next-generation sequencing (NGS), enables the rapid identification of species in environmental samples. Metabarcoding uses short gene sequences in order to identify species. It proven to be fast and cost effective [12, 13], making it possible to track and measure biodiversity over vast areas and time spans [14, 15]. One of the great advantages of metabarcoding is that rare species may be detected with greater sensitivity [16]. This is one of the reasons why NGS-based methods are being increasingly used to detect invasive species [17]. Moreover, DNA metabarcoding allows for identification of species that cannot be discriminated based on morphological features. However, we currently know little about the reliability of this method, and as yet there are no clear methodological guidelines regarding how it should be used. Therefore, in order to evaluate how accurate NGS is when it comes to estimating microorganism diversity, comparisons with morphology-based analysis need to be made. Many studies use pre-defined communities in order to verify their data e.g. [18–20]. The usage of mock communities may indeed improve the metabarcoding data validation process, but it has some shortcomings. Most mock communities often contain a lower number of species than field samples, and the quality of the DNA is often higher. Only a few rigorous studies have compared molecular versus morphology-based methods using samples from the same sampling sites, particularly with regard to microalgal diversity [21, 22]. Furthermore, the results from these studies are inconclusive. In a paper by Abdad et al. [21] similar spatial and temporal trends of taxonomic diversity were found for metabarcoding and microscopic studies of zooplankton, but not for phytoplankton. Moreover, they found that DNA metabarcoding could have the potential for semi-quantitative assessment of organisms’ abundances as well. Conversely, Zimmermann et al. [22] found no correlation between the number of reads obtained by NGS sequencing and the number of cells counted in a light microscope.

We here describe a mesocosm experiment to investigate the potential of using DNA metabarcoding to gain primary producer diversity and composition estimates. Previous studies have demonstrated that consumer species richness may increase the number of primary producer species due to complementary effects [23, 24]. We therefore designed the mesocosm experiment to test how consumer species richness influences primary producer species richness. A clear alteration of primary-producer community composition due to consumer species grazing is expected to be reflected by both morphological and molecular methods.

We allowed two species of benthic grazers (a gastropod and a mayfly larva) belonging to two different functional feeding groups, to feed upon natural, benthic microalgae-dominated biofilms (periphyton) over a period of 50 days. We sampled the biofilms for molecular (Roche 454 sequencing) and morphology-based analyses, and compared the abundances and diversity estimates generated from the two methods. We find great discrepancies between the methods; the NGS approach provided us with valuable information regarding species richness, whereas the morphology-based method provided species abundance and biodiversity measurements.

## Material and methods

The experiment was conducted in freshwater mesocosms on campus at the University of Cologne, NW Germany. Algal biofilm was pre-grown on tiles (4.7 x 4.7 cm) for two months in tanks with WC medium [25] based on an inocolum from a nearby pond. Two months later, when a thick algal biofilm had developed, the tiles were divided between 12 ten-liter buckets (two tiles in each bucket), each filled with 5 L of water. A net was placed over the buckets to prevent other potential consumer species from feeding upon the algae. The treatments consisted of a control treatment without consumer species, a treatment with juvenile *Lymnaea stagnalis* gastropods, a treatment with *Cloeon dipterum* mayfly larvae, and a treatment with both consumer species; each replicated threefold (S1 Table). The consumer species were collected from the same pond from which the water was taken. The consumer species were not endangered nor were they legally protected. All conditions for animal maintenance and experiments were carefully optimized to ensure that they met the animals’ needs. A specific ethical approval by the university’s Institutional Animal Care and Use Committee is not required for work with invertebrates according to German law. Regardless, we undertook all necessary measures to minimize animal suffering and followed guidelines for the use of animals in research and teaching activities [26]. We estimated the fresh weight of the consumer species to ensure that it did not differ between treatments. The experimental units were checked daily for emerging *C*. *dipterum*. In case of emergence, *C*. *dipterum* larvae were replaced. The animals were weighed every three days to ensure the same fresh weight (± 5 mg) in all treatments. If the fresh mass ratios deviated, animals were either removed or added to ensure the same fresh mass of consumers in each enclosure. After 50 days, the experiment was terminated. Samples were taken for dry mass, as well as for morphological and molecular determination of algal community composition.

### DNA extraction, PCR and sequencing

DNA was isolated using DNeasy Blood and Tissue Kits (Qiagen, Netherlands, S1 Fig). 200 μl of buffer ATL and 20 μl of proteinase K were added to the samples. The samples were then homogenized with Minilys (Bertin instruments, France) using Precellys ceramic kit 1.4/2.8 mm (Bertin instruments, France) for 2 x 20 sec at 5000 rpm. The samples were incubated for 10 min at 56°C in a thermomixer and then centrifuged for 3 min at 17000 x g. The subsequent steps were conducted in accordance with the instructions of the manufacturer of the kit. The sample was incubated for 1 min at room temperature and then centrifuged for 1 min at 6000 x g. The DNA was stored at −20°C until further processing.

The DNA concentration was estimated and purity was verified in a NanoDrop spectrophotometer (Implen, Germany) at 260 and 280 nm. DNA purity and quantity were also verified by electrophoresis in 1.2% agarose gels on 1 x TAE buffer stained with 1 x GelRed^TM^ (Biotium Inc., USA). The samples’ DNA content was adjusted to 25 ± 3 ng/μl prior to PCR amplification. The hypervariable V4 region of the 18S of the rRNA genes was amplified using the forward primer 5’—AATTCCAGCTCCAATAGCGTATAT—3’ and the reverse primer 5’—TTTCAGCCTTGCGACCATAC—3’. The primer pair was designed to ensure amplification of both green algae and diatoms. 500 random algae 18S rDNA sequences were aligned in Geneious [27], after which Primer3 [28] was used to find the best primer sites. NetPrimer (PREMIER Biosoft International, Palo Alto, CA) was used to further optimize the design of the primer pair. To ensure sample recognition in downstream analyses, the samples were amplified with tagged primers–the forward primer was tagged specifically for each sample using unique 10 bp tags (MID sequences). For all primers, the GS FLX Shotgun adaptors were attached in order to make them compatible with pyrosequencing procedures. The 25 μl PCR assay comprised of 25 ng of DNA, 0.02 U/μl Phusion Green Hot Start II High-Fidelity DNA Polymerase (Thermo Fisher Scientific, USA), 0.5 μM of each primer, 5 μl of 5x Phusion HF Buffer (Thermo Fisher Scientific, USA), 200 mM dNTP mix (VWR International, USA) and 15.75 μl of ultrapure water. PCR amplifications were carried out using FlexCycler (Analytik Jena, Germany). PCR cycling parameters consisted of an initial denaturation step at 98°C for 30 s, followed by 30 amplification cycles of 98°C for 10 s, 59°C for 30 s, 72°C for 30 s and 10 min final extension at 72°C. In order to reduce the effect of PCR biases that may have occurred in any given reaction, each of the samples were PCR-amplified three times. The PCR products were purified using the GenElute™ PCR Clean-Up Kit (Sigma-Aldrich, USA) following the manufacturer’s protocol. The DNA concentration of the samples was again estimated using a NanoDrop spectrophotometer, adjusted to 25 ± 3 ng/μl and pooled. The ready-to-load library was then sent to GATC Biotech (Germany) for 454 GS FLX paired-end sequencing (Roche Applied Science).

### Sequence analysis

We amplified approximately 500–600 bp within the 18S gene, including the hypervariable V4 region [29, 30] (S1 Fig). Mothur v. 1.34.4 [31] was used to filter the raw data. First the FASTQ files were converted to FASTA and QUAL files, and the sequences were trimmed using the trim.flows command in Mothur (pdiffs = 0, bdiffs = 0). After this step, all sequences shorter than 200 bp were omitted (mintlength  =  200), together with sequences with homopolymers longer than 8 bp (maxhomop  =  8). No ambiguous base calls were allowed (maxambig  =  0). Approximately 70% of all sequences remained after these steps. The resulting reads were dereplicated (collapsed to unique sequences) using the unique.seqs command and aligned to the SILVA reference alignment, v. 123, [32] with the align.seqs command. We then ran the screen.seqs command in Mothur to ensure that all sequences overlapped in the same alignment space. Approximately 20% of all sequences remained after this step. Thereafter, all sequences that are within 2 bp of a more abundant sequence were clustered. Chimeras were detected using UCHIME [33] with the chimera.uchime command. OTUs (operational taxonomic units) were built using the dist.seqs command in Mothur (cutoff = 0.15). Reads were clustered with sequence divergence threshold at 1% with the cluster command and singletons were discarded. For taxonomic annotation, the resulting sequences were imported into Geneious [27], where we performed a local MegaBLAST [34] search of each OTU versus a local DNA reference library with gap costs set to linear and with a match score of 1 and mismatch score of -2. The local reference databases were constructed based on the PR^2^ database [35]. The best MegaBLAST hit against our local database was used to classify each sequence, and a positive identification was defined as a hit with at least 90% identity and 100% query coverage. The OTUs were not assigned to the closest hit in the database; we instead based the final taxonomic determination upon the pairwise identity to the reference sequence to avoid erroneous identification. Reads with a 99, 97 and 90% pairwise identity were assigned to species, genus, or family, respectively [36]. The rarefaction curves were constructed using the rarefaction.single command (calc = sobs, freq = 100). To construct the phylogenetic tree, we used the dist.seqs command in Mothur. With the dist.seqs command the uncorrected pairwise distances between aligned DNA sequences are calculated. By default, a gap is penalized once and terminal gaps are penalized. We then ran the clearcut program (http://bioinformatics.hungry.com/clearcut/) within Mothur, using the clearcut command. Clearcut required the distance matrix created by the dist.seqs command. The clearcut program uses relaxed neighbor joining (RNJ) algorithm [37] when constructing phylogenetic trees. By running the clearcut command a file called abrecovery.tre is generated. Finally, we uploaded the abrecovery.tre file to the Interactive Tree of Life (iTOL) tool [38] in order to illustrate the taxonomic community compositions via a phylogenetic tree.

### Morphology-based analysis

Benthic algae were counted in three subsamples of each Lugol-preserved sample (diluted 1:32 with tap water) using an inverted microscope at a magnification of 400–1000x. In order to obtain the total biovolume of the species in each sample, the average number of cells per taxon was multiplied with the biovolume estimates, based on the typical cell morphology [39]. The taxonomic identity was determined to the lowest possible level; some taxa were grouped into non-taxonomical groups where the method did not allow for higher taxonomic resolution.

### Statistics

Before the statistical tests, all data were checked for homoscedasticity using Levene’s test. All statistical tests were conducted in SigmaPlot (v.11, SysStat). A one-way ANOVA was conducted to test for differences in algal dry mass between the grazing treatments, with algae dry mass as dependent variable and the grazing treatments as independent variable. A two-way ANOVA tested for the grazing effect upon single taxa followed by *post-hoc* comparisons with Tukey’s HSD. Here the treatment and algae species were independent variables and the number of cells was dependent variable. The data were ranked in MS Excel prior to the two-way ANOVA due to heteroscedasticity. Linear regressions were conducted to test for correlations between abundances of single taxa as determined by both methods.

## Results

### The morphology-based approach

18 primary producer taxa were identified microscopically (Table 1): 2 were identified to species level and 11 to genus level. We found one euglenoid, two cyanobacteria, two diatom, and twelve green algal taxa. The biovolume per cell differed strongly between taxa, ranging from 5 to 11,665 μm^3^ per cell.

**Table 1:** Primary producers found in the biofilm communities determined under the light microscope, given together with the geometric shapes of their cells and the cell-specific biovolume as calculated on basis of geometric figures according to [39].

Algae groups	Taxa	Geometric shape approximation	Average biovolume (μm^3^)
Cyanobacteria	*Anabaena* sp.	Cylinder, two half spheres	85
Cyanobacteria	*Pseudanabena*, *cf*	Cylinder, two half spheres	10
Diatoms	Diatoms, ribbon colonies	Box	260
Diatoms	Diatoms, solitary	Prism on elliptic base	150
Green algae	*Chlamydomonas*, *cf*	Prolate spheroid	675
Green algae	*Closterium* sp.	Double cone	7070
Green algae	Coccoid green algae	Sphere	60
Green algae	*Cosmarium* sp.	Double spheroid	11665
Green algae	*Monoraphidium* sp.	Double cone	75
Green algae	*Oedogonium* sp.	Cylinder	435
Green algae	*Ooystis* sp.	Prolate spheroid	250
Green algae	*Pediastrum* sp.	Box	310
Green algae	*Scenedesmus* sp., two celled colonies	Prolate spheroid	35
Green algae	*Scenedesmus* sp., four celled colonies	Prolate spheroid	9
Green algae	*Selenastrum* sp.	Double cone	5
Green algae	*Tetraedon caudatum*	Box	65
Green algae	*Tetraedon minimum*	Box	185
Euglenoids	*Trachelomonas* sp.	Sphere	2145

Based on cell numbers, *Pseudanabena* sp. was the dominant primary producer, while *Cosmarium* sp. was dominant in terms of biovolume due to their large cell size (S2 Fig; Table 1).

### The molecular approach

**Fig 1:**
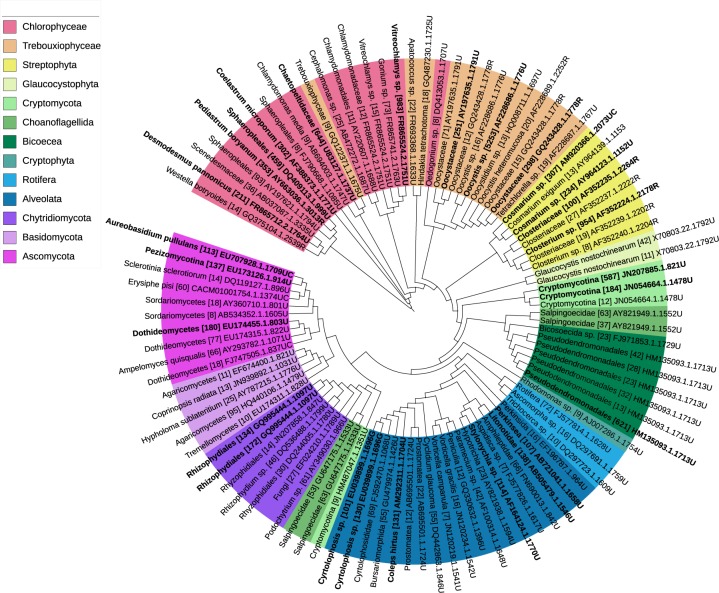
Phylogenetic tree based upon the 100 most abundant OTUs. OTUs with more than 100 sequences are market in bold. The number of OTUs obtained is displayed in parenthesis. The OTUs are clustered at 99% identity and blasted against the PR^2^ reference database for taxonomic identification. The phylogenetic tree was constructed by the relaxed neighbor joining algorithm in the program Clearcut and visualized using iTOL.

We obtained 139,702 sequences, of which 15,662 sequences remained after quality filtering. Clustering at 99% identity yielded 195 OTUs (Fig 1). The number of reads per sample after quality filtering varied from 325–2,393 (S3 Fig). The majority of the sequences were classified as fungi (36%), followed by algae (33%), ciliphora (15%), bicoecea (5%) and choanoflagellida (4%). 64 OTUs were identified as algae. The algal OTUs were identified at different taxonomic levels: 13 to species (20%), 25 to genus (39%), 20 to family (31%) level. The remaining six OTUs could only be determined at the level of order due to a lack of higher resolution in the reference database.

### Comparison of the morphology-based and the molecular approach

**Fig 2:**
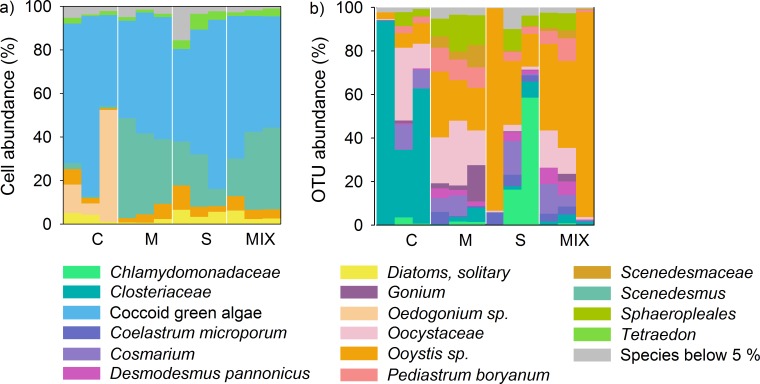
The species composition from morphology-based (a) and molecular (b) data of all twelve samples. The two species of *Tetraedon*, identified with the morphology-based method, were grouped and cyanobacteria were removed (as they cannot be detected with the 18S primer set for eukaryotes) for a better comparison. The biofilms were subjected to grazing by *C*. *dipterum*, *L*. *stagnalis* or by both consumer species over a period of 50 days. No consumer species were present in the control treatment. The four treatments are labeled C = Grazer-free control, M = *C*. *dipterum* (Mayfly), S = *L*. *stagnalis* (Snail), MIX = *C*. *dipterum* and *L*. *stagnalis*.

Eight algal taxa were detected using both the molecular and the morphology-based approach (S3 Table). *Monorhaphidium* sp., *Selenastrum* sp., *Trachelomonas* sp. and the cyanobacteria were not identified by the metabarcoding approach (S3 Table). Moreover, the algal community composition varied strongly between the molecular and the morphology-based approach (Fig 2). Based upon the morphological data (excluding the cyanobacteria), coccoid green algae were the most abundant group of primary producers (58%), followed by *Scenedesmus* sp. (22%). In comparison, the molecular approach yielded *Oocystaceae* as the most abundant group of primary producers (45%) followed by *Closteriaceae* (18%).

**Fig 3:**
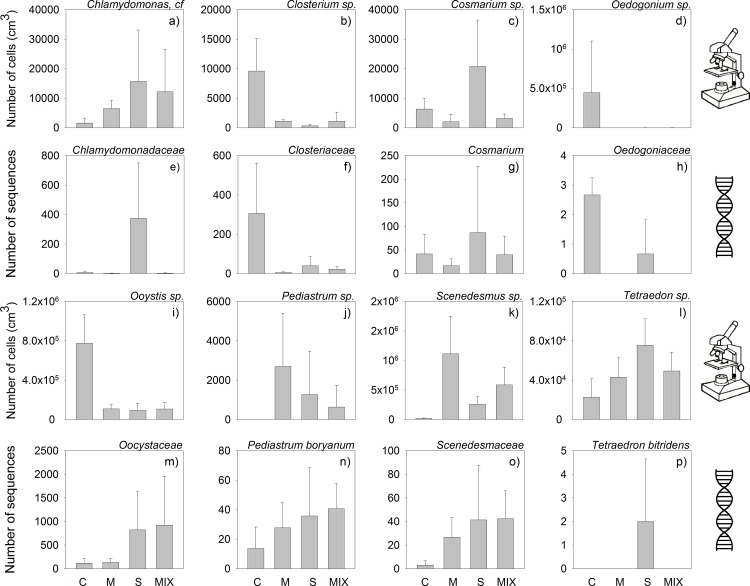
Comparisons of grazing impact on single algal taxa based upon microscopic identification (a—d, i—l) and sequence data (e—h, m—p). The biofilms were subjected to grazing by *C*. *dipterum*, *L*. *stagnalis*, or by both consumer species over a period of 50 days. No consumer species were present in the control treatment. The four treatments are labeled C = Grazer-free control, M = *C*. *dipterum* (Mayfly), S = *L*. *stagnalis* (Snail), MIX = *C*. *dipterum* and *L*. *stagnalis*. Bars represent algal cell (a—d, i—l) and sequence numbers (e—h, m—p, mean ± SD, N = 3).

We conducted linear regressions between the abundances of taxa across all treatments identified using both the molecular and the morphology-based methods (Fig 3, Table 2), and found significant correlations between the morphology-based and molecular abundance data for *Closterium* sp. (Fig 3B and 3F), *Cosmarium* sp. (Fig 3C and 3G), and *Oedogonium* sp. (Fig 3D and 3H, Table 2).

**Table 2:** Results of linear regressions (F-statistics and P-values; d.f. = degrees of freedom) between the abundances of specific taxa obtained via the morphology-based and the molecular method.

Algae taxa	Equation	df	F	P
*Chlamydomonas sp*.	y = 1.06 + (0.03x)	3	2.017	0.291
*Closterium sp*.	y = -3.64 + (0.032x)	3	62.349	**0.016**
*Cosmarium sp*.	y = 19.815 + (0.00326x)	3	25.082	**0.038**
*Oedogonium sp*.	y = 0.220 + (0.00000548x)	3	30.249	**0.032**
*Ooystis sp*.	y = 704.959–(0.000763x)	3	1.086	0.407
*Pediastrum sp*.	y = 26.437 + (0.00259x)	3	0.138	0.746
*Scenedesmus sp*.	y = 21.458 + (0.0000139x)	3	0.295	0.642
*Tetraedron sp*.	y = -1.365 + (0.0000391x)	3	5.348	0.147

### Grazing effects

The consumer species varied significantly in their feeding preferences, resulting in distinct algal community composition changes (S4 Fig; S2 Table). We found a highly significant interaction between the grazing treatments and the cell number of particular primary producer species (Table 3).

**Table 3:** Two-way ANOVA on the effect of consumer species combination (treatment) on the cell number of single algal taxa. F-statistics and P-values are shown. d.f. = degrees of freedom.

Effect	df	F	P
Algal taxa	16	0.85	0.63
Treatment	3	0.91	0.44
Algal taxa x Treatment	48	4.37	0.000

For 13 of 18 primary producer species, significant grazing effects were found upon cell number abundances (S4 Fig). For example, the morphology-based approach revealed a higher abundance of *Closterium* sp. and *Oedogonium* sp. in the control treatment when compared to the grazer treatments (Fig 2; S4 Fig). Similarly, the DNA metabarcoding approach found a high abundance of *Closteriaceae* in the control treatment in comparison to the grazer treatments (Fig 2), however very few *Oedogonium* sp. sequences were detected. Still, the DNA metabarcoding displayed a greater variation between the OTU richness and abundances between the treatments when compared to the morphology-based approach (Fig 2). The biological replicates analyzed using the both the DNA metabarcoding approach and the morphology-based approach were highly consistent, indicating that both methods provide stable and reliable results (Fig 2). Yet, we could not find a significant correlation between consumer species richness and primary producer using neither the morphology-based (y = 14.500 –(0.167x), R^2^ = 0.02, P = 0.67; S6A Fig)) nor the DNA metabarcoding approach (y = 20.500 + (2.333x), R ^2^ = 0.04, P = 0.51; S6B Fig)).

## Discussion

This study represents one of the first comparative analyses of algal community structure employing both morphology-based and molecular methods to investigate environmental samples originating from the same sampling areas. Metabarcoding uncovered a vast taxonomic diversity, largely exceeding the 18 taxa yielded by the morphology-based survey. Without any additional efforts (or identification expertise), the molecular approach identified additional eukaryotic taxa from the fungal and animal kingdoms. In total, 195 eukaryotic OTUs were detected in the DNA-based data set. Similarly, other studies investigating phytoplankton communities revealed a far greater species richness when using metabarcoding [21]. However, many metabarcoding studies have been criticized for misclassification of erroneous OTUs to new species (due to PCR and sequencing errors), which thereby may cause an overestimation of species diversity [40–42]. Therefore, any observations of low-abundance taxa need to be evaluated critically. Moreover, the intra- and interspecific diversity may greatly vary between species [43]. Consequently, the usage of a clustering divergence threshold may lead to an over and/or underestimation of the species richness. Here, the DNA metabarcoding approach covered most of the species found with the morphology-based approach, but not all. *Monoraphidium* sp., *Selenastrum* sp. and *Trachelomonas* sp. were not identified by the metabarcoding, despite the fact that all three genera are included in the reference database. This may be explained by erroneous morphological classification, or misclassification of OTUs due to low variability within the metabarcoding marker.

We were only able to identify 59% of our OTUs to genus level. This is within the range of previous studies performed on unicellular eukaryotes [15, 21]. The lack of complete and curated DNA reference libraries is one of the limiting factors for the identification of species in metabarcoding studies [44, 45]. If DNA metabarcoding is to become a standard alternative or supplement to morphology-based approaches, we need to fill gaps in the DNA reference databases, which in turn is impossible without comprehensive taxonomic expertise. To increase the level of taxonomic identification of our sequences, we choose to use the PR^2^ database [35]. This is a database specializing in 18S rDNA sequences of protists, with the benefit of being curated.

Metabarcoding is emerging as a promising method in biodiversity research. Yet, the lack of rigorous frameworks for analyzing NGS data makes comparisons between studies difficult. Among other factors, the taxonomic resolution in DNA metabarcoding studies depends heavily on the selection of the primer pairs. We designed a primer pair to ensure amplification of green algae and diatoms, targeting the v4 region in the 18S rDNA gene. As the 18S rDNA gene is a highly conserved gene found in all eukaryotes, this allowed us to analyze a broad taxonomic spectrum of the eukaryotic diversity. Universal primer pairs were shown to amplify only half of the OTUs revealed when using more selective primer pairs [46]. The usage of a specific primer set may have resulted in an increased OTU richness. However, universal primer pairs (e.g. the 18S rDNA gene) are more often included in reference databases [32, 35], the use of a more selective primer pair may therefore have reduced the number of identified OTUs.

Due to the coarse scale of the taxonomic identification of algae species provided by the morphology-based approach, we were not able to compare some of the common groups identified with the DNA metabarcoding results (e.g. coccoid green algae). Nevertheless, we found that the abundance pattern of the NGS data clearly differed from that of the morphology-based analysis. Based upon the molecular data, the second most common algal family (after *Oocystaceae*) was *Closteriaceae*, which comprised less than two percent of the primary producer community according to the morphology-based analysis. These deviations in the abundance data between the morphology-based and molecular method may have been caused by copy number variation [47, 48], pseudogenes [49] and/or number of nuclei per individual. Copy number of the rDNA gene can vary dramatically across taxa [47] and even within species [50]. While copy number variation is found in archaea and bacteria [51], it is far more extensive for eukaryotes, where up to tens of thousands copies haven been found [47]. Moreover, DNA extraction [52] and PCR amplification [53] are also known to influence abundance estimates.

Even if there is a bias in the PCR procedure, DNA extraction and interspecific copy number variation, we may still be able to compare the abundance of single species between treatments. With respect to *Closterium* sp. and *Oedogonium* sp., the abundance of the algal species was significantly reduced in the consumer species treatments when compared to the control in both morphology-based and molecular-based approaches. Nevertheless, the molecular and morphology-based abundance data only correlated in 37.5% of the cases. This means that in the majority of the cases, the outcome of the abundance data differed between methods. Similar results have been found regarding pollen [54], plants [55], nematodes [56], macroinvertebrates [57], diatoms [18] and phytoplankton [21].

### Grazing effects

It was obvious from both methodological approaches that the algal communities were strongly impacted by invertebrate grazing activity. With regards to the morphology-based data, we found that the abundance of *Oedogonium* sp. (a filamentous green algae) and diatoms (ribbon colonies) decreased in the presence of grazers. Both *Radix peregra* (a close relative to *L*. *stagnalis*) and *C*. *dipterum* were shown to prefer filamentous algae by previous studies [58, 59]. The cell abundances of *Closterium* sp., the second largest algae taxa identified, decreased in the presence of grazers. This results confirms previous findings, where snails [60–62] and mayfly larvae [59] were shown to prefer larger algal cells. We also identified grazer-specific effects. *C*. *dipterum* seemed to prefer *Trachelomonas* sp., whereas *L*. *stagnalis* ingested a higher proportion of *Scenedesmus* sp. Conversely, *Monoraphidium* sp., *Oocystis* sp., *Pediastrum* sp., and *Selenastrum* sp. decreased with grazer absence, likely caused by an increased competition between the algae species.

We expected a positive linear correlation between consumer species richness and primary producer richness, but there was no statistically significant result confirming this. However, with the DNA metabarcoding approach, we found a non-significant increase of primary producer richness with increasing grazer richness as expected, suggesting that the DNA metabarcoding approach may be more sensitive in detecting environmental changes.

## Conclusions

Although considerable progress has recently been made in DNA metabarcoding, many challenges remain. Additional steps need to be taken to promote clearer methodological guidelines for analyzing NGS data. The methods and results reported herein contribute to the development of a fast, reliable and cost effective way to analyze algae communities. In conclusion, using DNA metabarcoding together with morphology-based traditional methods when assessing algal biodiversity increases the reliability of the outcomes. Species richness estimates can be made if careful measures are taken to avoid the overestimation of taxa from sequencing errors. DNA metabarcoding is a useful tool when it comes to detecting rare taxa and overall changes in community compositions, however, without comprehensive curated reference libraries, DNA metabarcoding lacks the power to contribute to algal species richness estimates.

## Supporting information

S1 Fig Workflow of experimental pipeline. The major bioinformatics steps of the experimental pipeline.
(TIF)

S2 Fig The average primary producer community composition (including cyanobacteria) based on cell number (a) and biovolume (b), determined microscopically in relation to the consumer treatment: C = Grazer-free control, M = *C*. *dipterum* (Mayfly), S = *L*. *stagnalis* (Snail), MIX = *C*. *dipterum* and *L*. *stagnalis*.
(TIF)

S3 Fig Rarefaction curve of all OTUs clustered at 99% identity in the four grazer treatments: C = Grazer-free control, M = *C*. *dipterum* (Mayfly), S = *L*. *stagnalis* (Snail), MIX = *C*. *dipterum* and *L*. *stagnalis*. (TIF)

S4 Fig Impact of grazing on single algal species based on microscope counts. Bars represent mean (± SD) algal cell numbers in the different treatments: C = Grazer-free control, M = *C*. *dipterum* (Mayfly), S = *L*. *stagnalis* (Snail), MIX = *C*. *dipterum* and *L*. *stagnalis*. Means that were found to be significantly different after *post-hoc* comparisons are labeled with superscript letters. Only taxa where significant grazing effects were found are displayed.
(TIF)

S5 Fig Mean (± SD of n = 3) algal dry mass dependent on the consumer treatment: C = grazer-free control, M = *C*. *dipterum* (Mayfly), S = *L*. *stagnalis* (Snail), MIX = *C*. *dipterum* and *L*. *stagnalis*. One-way ANOVA (d.f. = 11, F = 3.31, P = 0.08, N = 3).
(TIF)

S6 Fig The effect of consumer species richness on primary producer richness. Primary producer species richness a) and OTU richness b) (N = 3) after grazing by 0–2 consumer species over a period of 50 days. The results of the linear regression are represented as a solid line. The four treatments are labeled C = Grazer-free control, M = *C*. *dipterum* (Mayfly), S = *L*. *stagnalis* (Snail), MIX = *C*. *dipterum* and *L*. *stagnalis*.
(TIF)

S1 Table Experimental setup. To ensure equal grazing pressure in all units, equal fresh weight of the consumer species were added.
(DOCX)

S2 Table P-values derived from Tukey post-hoc comparisons regarding the effect of grazing on the cell number of single algal taxa in the treatments: C = Grazer-free control, M = *C*. *dipterum* (Mayfly), S = *L*. *stagnalis* (Snail), MIX = *C*. *dipterum* and *L*. *stagnalis*). (DOCX)

S3 Table The primary producer species detected using the morphology-based method and the molecular method, and taxa detected using both methods. If taxa were detected at higher taxonomic rank using the alternative method, the higher taxonomic rank is noted up to family level.
(DOCX)
