# Supplementary figures and images for: The best of both worlds: A combined approach for analyzing microalgal diversity via metabarcoding and morphology-based methods
Source: PLoS One. 2017 Feb 24;12(2):e0172808. doi: 10.1371/journal.pone.0172808 (PMC5325584; doi:10.1371/journal.pone.0172808)

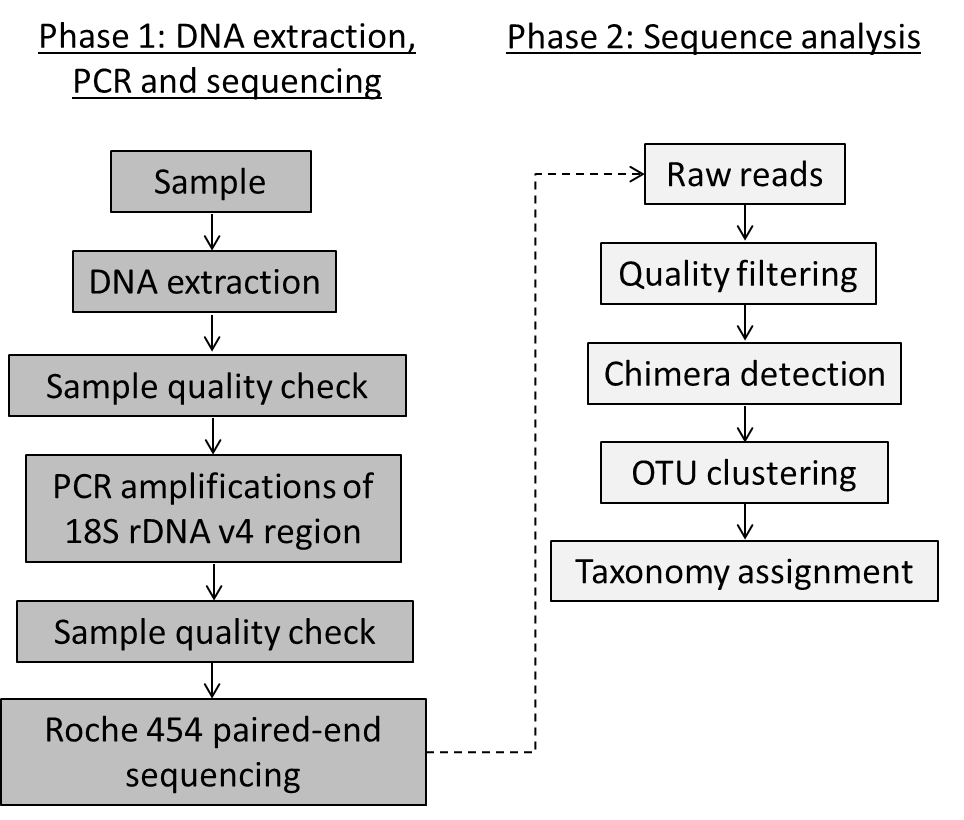

Supplement: S1 Fig — The major bioinformatics steps of the experimental pipeline. (TIF) [file pone.0172808.s001.tif]

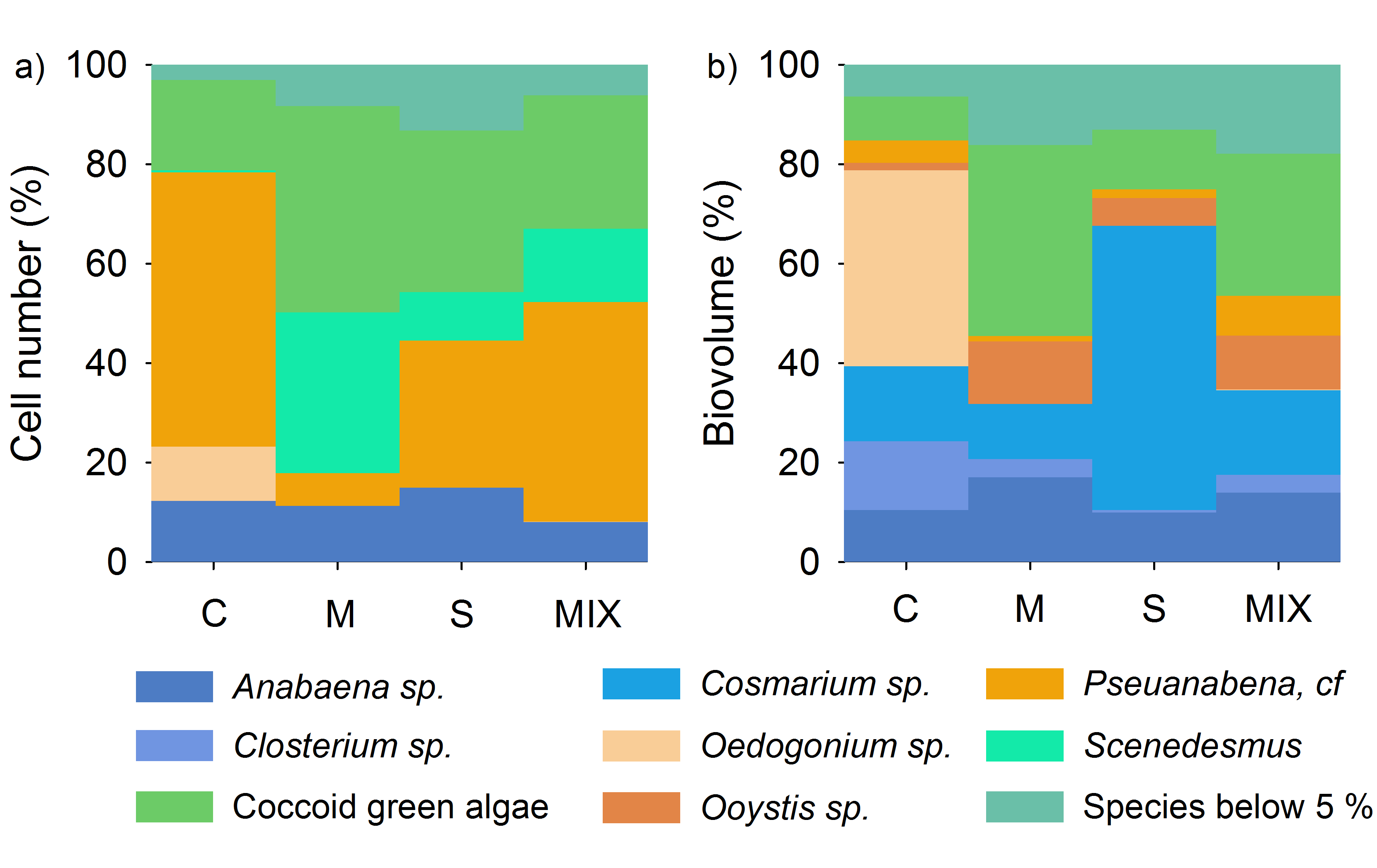

Supplement: S2 Fig — The average primary producer community composition (including cyanobacteria) based on cell number (a) and biovolume (b), determined microscopically in relation to the consumer treatment: C = Grazer-free control, M = C. dipterum (Mayfly), S = L. stagnalis (Snail), MIX = C. dipterum and L. stagnalis. (TIF) [file pone.0172808.s002.TIF]

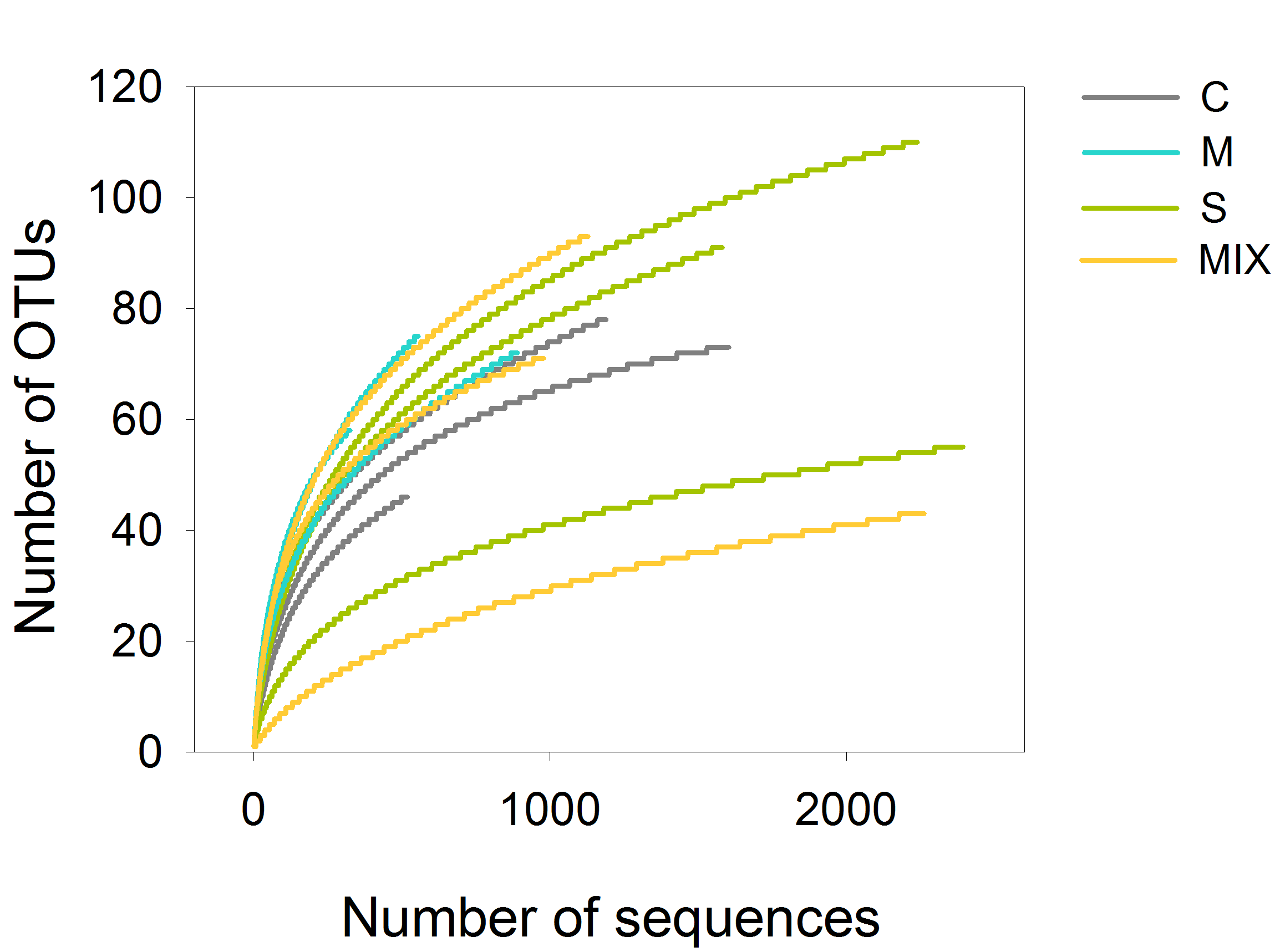

Supplement: S3 Fig — (TIF) [file pone.0172808.s003.TIF]

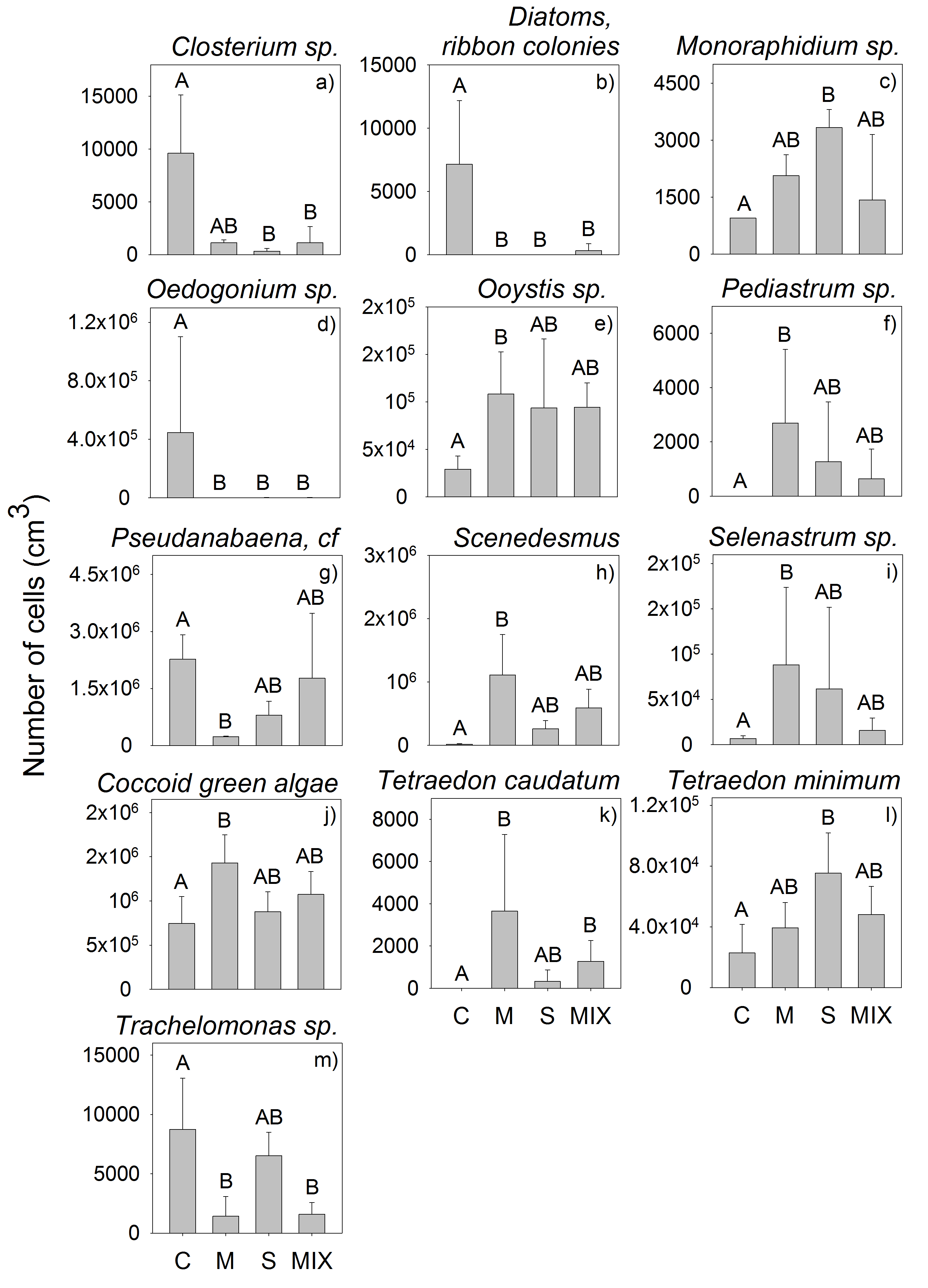

Supplement: S4 Fig — Bars represent mean (± SD) algal cell numbers in the different treatments: C = Grazer-free control, M = C. dipterum (Mayfly), S = L. stagnalis (Snail), MIX = C. dipterum and L. stagnalis. Means that were found to be significantly different after post-hoc comparisons are labeled with superscript letters. Only taxa where significant grazing effects were found are displayed. (TIF) [file pone.0172808.s004.TIF]

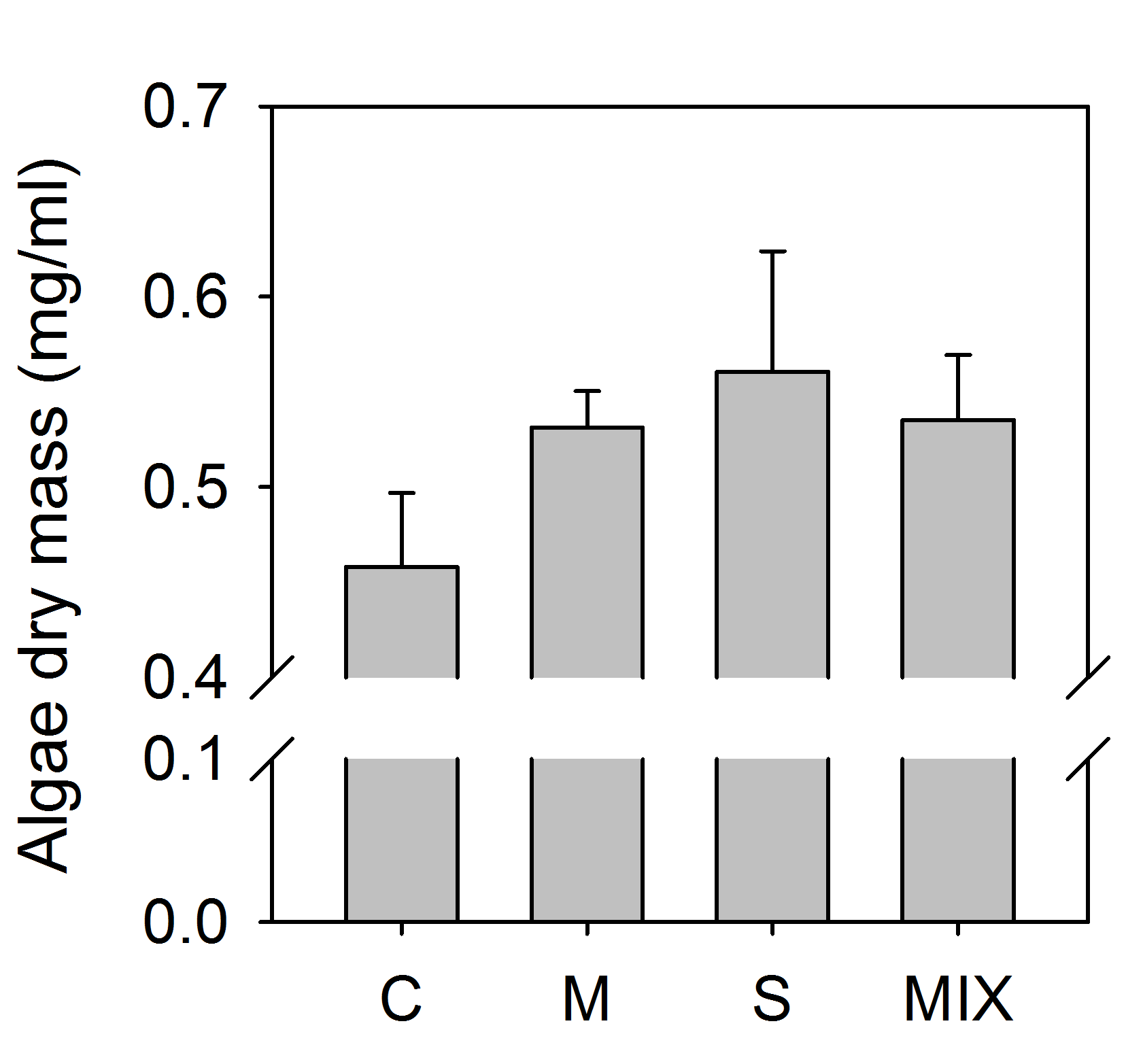

Supplement: S5 Fig — One-way ANOVA (d.f. = 11, F = 3.31, P = 0.08, N = 3). (TIF) [file pone.0172808.s005.TIF]

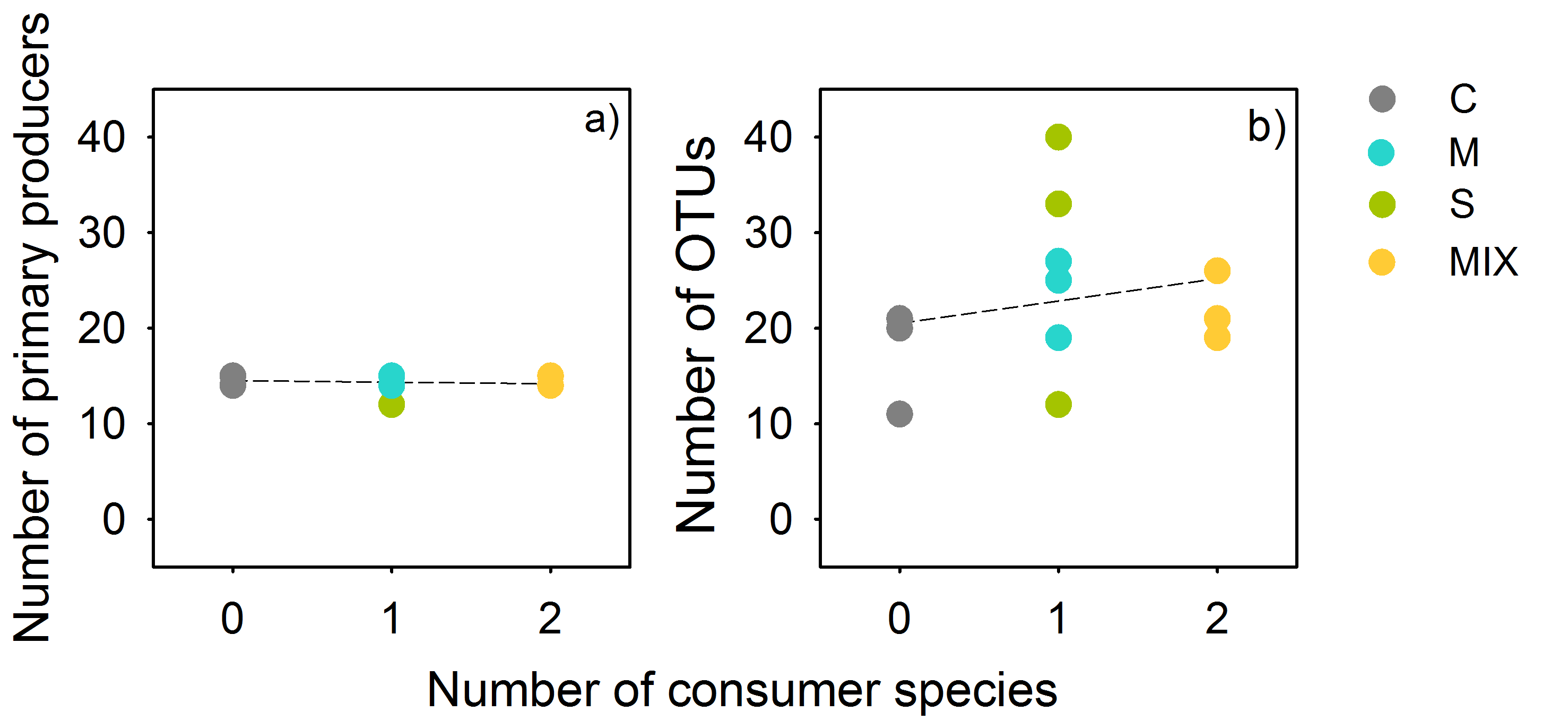

Supplement: S6 Fig — Primary producer species richness a) and OTU richness b) (N = 3) after grazing by 0–2 consumer species over a period of 50 days. The results of the linear regression are represented as a solid line. The four treatments are labeled C = Grazer-free control, M = C. dipterum (Mayfly), S = L. stagnalis (Snail), MIX = C. dipterum and L. stagnalis. (TIF) [file pone.0172808.s006.TIF]
