# Supplementary material for: The best of both worlds: A combined approach for analyzing microalgal diversity via metabarcoding and morphology-based methods
Source: PLoS One. 2017 Feb 24;12(2):e0172808. doi: 10.1371/journal.pone.0172808 (PMC5325584; doi:10.1371/journal.pone.0172808)
Supplement: S1 Table — To ensure equal grazing pressure in all units, equal fresh weight of the consumer species were added. (DOCX) [file pone.0172808.s007.docx]

| **Consumer species added** | **Number of consumer species** | **Replicates** |
| --- | --- | --- |
|  | 0 | 3 |
| *Cloeon dipterum* | 1 | 3 |
| *Lymnaea stagnalis* | 1 | 3 |
| *C. dipterum & L. stagnalis* | 2 | 3 |
