# Supplementary material for: The best of both worlds: A combined approach for analyzing microalgal diversity via metabarcoding and morphology-based methods
Source: PLoS One. 2017 Feb 24;12(2):e0172808. doi: 10.1371/journal.pone.0172808 (PMC5325584; doi:10.1371/journal.pone.0172808)
Supplement: S2 Table — (DOCX) [file pone.0172808.s008.docx]

| **P-value** | | | | | |
| --- | --- | --- | --- | --- | --- |
| **Treatment/**  **algae taxa** | *Closterium sp.* | *Coccoid* green algae | *Diatoms*, ribbon colonies | *Monorhaphidium sp.* | *Oedogonium sp.* |
| **C vs. S** | **0.00** | **0.03** | **0.000** | **0.00** | **0.000** |
| **C vs. MIX** | **0.02** | 0.07 | **0.000** | 0.05 | **0.01** |
| **C vs. M** | 0.25 | 0.48 | **0.01** | 0.40 | **0.01** |
| **M vs. S** | 0.25 | 0.57 | 0.67 | 0.19 | 0.67 |
| **M vs. MIX** | 0.75 | 0.75 | 0.67 | 0.75 | 1.00 |
| **MIX vs. S** | 0.83 | 0.99 | 1.00 | 0.75 | 0.75 |
| **Treatment/**  **algae taxa** | *Ooystis sp.* | *Pediastrum sp.* | *Pseuanabena, cf* | *Scenedesmus* | *Selenastrum sp.* |
| **C vs. S** | **0.03** | **0.00** | **0.00** | **0.00** | **0.01** |
| **C vs. MIX** | 0.89 | 0.05 | 0.40 | 0.19 | 0.25 |
| **C vs. M** | 0.99 | 0.10 | 0.75 | 0.75 | 0.67 |
| **M vs. S** | 0.07 | 0.48 | 0.07 | 0.05 | 0.19 |
| **M vs. MIX** | 0.97 | 0.99 | 0.94 | 0.75 | 0.89 |
| **MIX vs. S** | 0.19 | 0.67 | 0.25 | 0.40 | 0.57 |
| **Treatment/**  **algae taxa** | *Tetraedon caudatum* | *Tetraedon minimum* | *Trachelomonas sp.* |  |  |
| **C vs. S** | **0.00** | **0.01** | **0.01** |  |  |
| **C vs. MIX** | **0.03** | 0.19 | **0.02** |  |  |
| **C vs. M** | 0.89 | 0.48 | 0.94 |  |  |
| **M vs. S** | **0.02** | 0.32 | 0.05 |  |  |
| **M vs. MIX** | 0.19 | 0.94 | 0.10 |  |  |
| **MIX vs. S** | 0.75 | 0.67 | 0.99 |  |  |
