# Supplementary material for: The best of both worlds: A combined approach for analyzing microalgal diversity via metabarcoding and morphology-based methods
Source: PLoS One. 2017 Feb 24;12(2):e0172808. doi: 10.1371/journal.pone.0172808 (PMC5325584; doi:10.1371/journal.pone.0172808)
Supplement: S3 Table — If taxa were detected at higher taxonomic rank using the alternative method, the higher taxonomic rank is noted up to family level. (DOCX) [file pone.0172808.s009.docx]

| **Taxa detected with light micrsocopy** | **Taxa detected with metabarcoding** | **Taxa detected with both methods** |
| --- | --- | --- |
| *Anabaena* sp. | *Achnanthidium minutissimum* | *Chlamydomonas* sp. |
| *Chlamydomonas, cf* | *Amphibiocystidium ranae* | *Closteriaceae* |
| *Closterium* sp. | *Ankistrodesmus* sp. | *Cosmarium* sp. |
| Coccoid green algae | *Cryptophyceae* | *Oedogonium* sp. |
| *Cosmarium* sp. | *Cephalomonas* | *Ooystis* sp. |
| Diatoms, ribbon colonies | *Characiopodium* | *Pediastrum* sp. |
| Diatoms, solitary | *Chlamydomonadaceae* 1 | *Scenedesmus* sp. |
| *Monoraphidium sp.* | *Chlamydomonadaceae* 2 | *Tetraedon* sp. |
| *Oedogonium* sp. | *Chlamydomonadaceae* 3 |  |
| *Ooystis* sp. | *Chlamydomonadaceae* 4 |  |
| *Pediastrum* sp. | *Chlamydomonadaceae* 5 |  |
| *Pseudanabena, cf* | *Chlamydomonadaceae* 6 |  |
| *Scenedesmus* sp. | *Chlamydomonas media* |  |
| *Selenastrum* sp. | *Chlorellaceae* 1 |  |
| *Tetraedon caudatum* | *Chlorellaceae* 2 |  |
| *Tetraedon minimum* | *Choricystis* sp. |  |
| *Trachelomonas* sp. | *Closteriaceae* 1 |  |
|  | *Closteriaceae* 2 |  |
|  | *Closteriaceae* 3 |  |
|  | *Closteriaceae* 4 |  |
|  | *Closteriaceae* 5 |  |
|  | *Closteriaceae* 6 |  |
|  | *Closteriaceae* 7 |  |
|  | *Closteriaceae* 8 |  |
|  | *Coelastrum microporum* |  |
|  | *Cosmarium eiguum* |  |
|  | *Cosmarium* sp. |  |
|  | *Chlamydomonadales* |  |
|  | *Desmidiaceae* |  |
|  | *Desmodesmus pannonicus* |  |
|  | *Glaucocystis nostochinearum* 1 |  |
|  | *Glaucocystis nostochinearum* 2 |  |
|  | *Gonium* |  |
|  | *Hindakia tetrachotoma* |  |
|  | *Oedogoniaceae* |  |
|  | *Oedogonium* sp. |  |
|  | *Oocystaceae* 1 |  |
|  | *Oocystaceae* 10 |  |
|  | *Oocystaceae* 2 |  |
|  | *Oocystaceae* 3 |  |
|  | *Oocystaceae* 4 |  |
|  | *Oocystaceae* 5 |  |
|  | *Oocystaceae* 6 |  |
|  | *Oocystaceae* 7 |  |
|  | *Oocystaceae* 8 |  |
|  | *Oocystaceae* 9 |  |
|  | *Oocystidium* |  |
|  | *Oocystis heteromucosa* |  |
|  | *Oocystis* sp. |  |
|  | *Pediastrum boryanum* |  |
|  | *Protodesmus globulifer* |  |
|  | *Pseudomuriella* |  |
|  | *Radiococcaceae* |  |
|  | *Rhodomonas* sp. |  |
|  | *Scenedesmaceae* |  |
|  | *Scenedesmus* |  |
|  | *Sphaeropleales* 1 |  |
|  | *Sphaeropleales* 2 |  |
|  | *Sphaeropleales* 3 |  |
|  | *Sphaeropleales* 4 |  |
|  | *Tetraedron bitridens* |  |
|  | *Trebouiophyceae* |  |
|  | *Ulotrichales* |  |
|  | *Westella botryoides* |  |
